# Homozygous Familial Hypercholesterolemia: Luck Meets Opportunity Meets Knowledge

**DOI:** 10.1016/j.jaccas.2022.10.005

**Published:** 2022-11-08

**Authors:** Lisa Young, Emily E. Brown, Seth S. Martin

**Affiliations:** Ciccarone Center for the Prevention of Cardiovascular Disease, Division of Cardiology, Department of Medicine, Johns Hopkins University School of Medicine, Baltimore, Maryland, USA

**Keywords:** atherosclerosis, cholesterol, coronary artery disease, genetics, low-density lipoprotein cholesterol, monoclonal antibodies, ASCVD, atherosclerotic cardiovascular disease, HoFH, homozygous familial hypercholesterolemia, LDL-C, low-density lipoprotein cholesterol, PCSK9, proprotein convertase subtilisin-kexin type 9

## Abstract

This case report describes a 67-year-old African-American woman with homozygous familial hypercholesterolemia caused by 2 pathogenic variants in the *LDLR* gene. Initial surgical, pharmacological, and low-density lipoprotein apheresis interventions were insufficient; the addition of proprotein convertase subtilisin-kexin type 9 and angiopoietin-like 3 inhibitors lowered her low-density lipoprotein cholesterol to <70 mg/dL. (**Level of Difficulty: Advanced.**)

## Central Illustration

**Figure:**
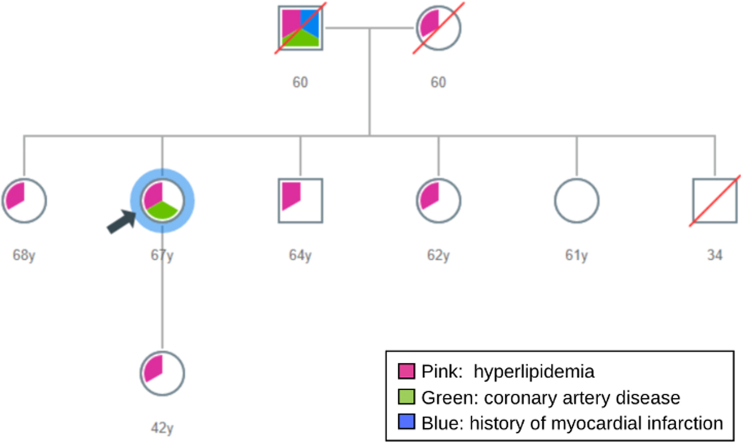

## History of Presentation

The patient is a 67-year-old African-American woman who presented with bilateral arcus and tendinous xanthomas at age 11 years. Her total cholesterol at this age was >1,000 mg/dL. Rapid xanthoma growth led to body image concerns and resection of elbow and knee xanthomas at age 12 years. A partial ileal bypass (PIB) then lowered her cholesterol to 608 mg/dL. She was discharged on a low-cholesterol diet. However, she experienced suicidal ideation caused by parental abuse and insufficient dietary support, subsequently becoming a ward of the state at age 13 years.

**Learning Objectives**

• To consider American Heart Association diagnostic criteria and integrate a patient’s genetic testing, family history, physical examination, and lipid panel into forming a differential diagnosis for HoFH.
• To understand the role of dietary, pharmacological, and surgical interventions in contributing to increased survival for HoFH patients. Early and aggressive intervention is essential to lower LDL-C to optimal levels (<100 mg/dL in adults without ASCVD; <70 mg/dL in ASCVD patients; <55 mg/dL in very-high-risk ASCVD patients) and prevent ASCVD and/or myocardial infarction.

**Figure 1:**
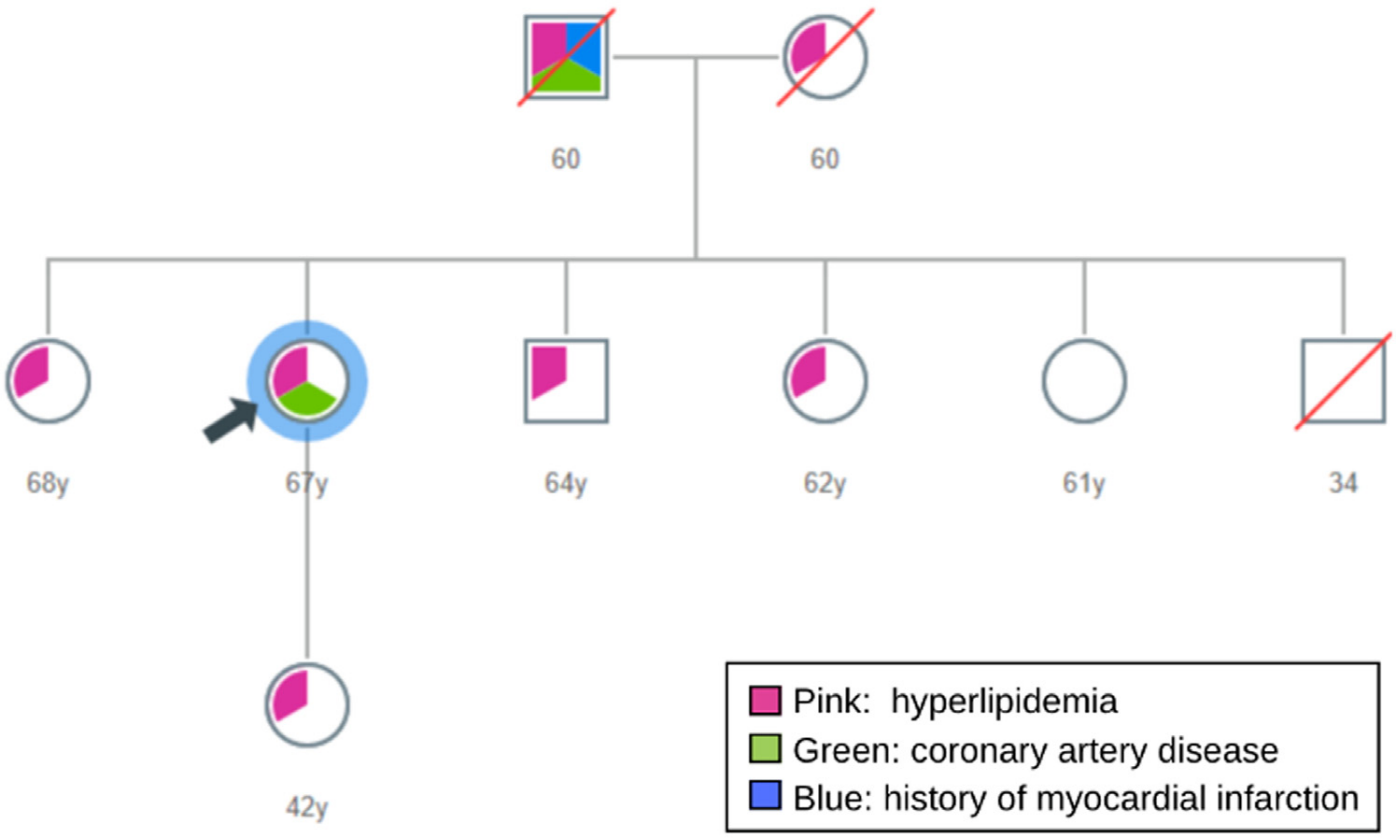
Pedigree The patient’s family history was significant for hyperlipidemia and cardiovascular disease.

Her father experienced arcus, hypercholesterolemia, and a fatal myocardial infarction at age 60 years; her mother had hypercholesterolemia (Figure 1). Of her 6 siblings, 3 have presumed heterozygous familial hypercholesterolemia. Her daughter, now age 42 years, has heterozygous familial hypercholesterolemia.

Two lipid-lowering agents, clofibrate and probucol 500 mg twice daily, were initiated at age 21 years but were discontinued because of rash development and the patient’s pregnancy at age 24 years, respectively. She developed severe congestive heart failure, which resolved postpartum. A bile acid sequestrant, cholestyramine 3 packs daily, was initiated but paused because of painful hemorrhoids.

At age 26 years, she was referred to our hospital’s lipid clinic and diagnosed with homozygous familial hypercholesteremia (HoFH). Cholestyramine 1 pack twice daily (then escalated to 4 packs daily) was restarted when her low-density lipoprotein cholesterol (LDL-C) levels reached the high 400s in mg/dL. Nicotinic acid 250 mg daily was initiated at age 29 years (subsequently increased to 1 g 3 times daily). Both nicotinic acid and cholestyramine were briefly paused following contraction of viral gastroenteritis. Cholestyramine was discontinued due to constipation. Lovastatin 20 mg twice daily (eventually increased to 40 mg twice daily) was prescribed at age 32 years.

At age 33 years, she developed exertional chest pain with preserved left ventricular ejection fraction on echocardiogram. Cardiac catheterization revealed 3 occlusions: 100%, right coronary artery; 99%, first diagonal branch of the left anterior descending artery; and 40%, left circumflex artery. Antianginal treatment improved her symptoms.

Plasmapheresis and lipoprotein apheresis were initiated at age 34 and 40 years, respectively. After each session, her LDL-C decreased from ∼500 to 60 mg/dL; the cumulative average LDL-C was still suboptimal. Lovastatin was switched to atorvastatin 80 mg daily at age 42 years, and evolocumab 420 mg monthly was added at age 61 years. Apheresis was discontinued at age 62 years because of vascular access challenges and evolocumab efficacy. At age 66 years, ezetimibe 10 mg daily was initiated.

**Figure 2:**
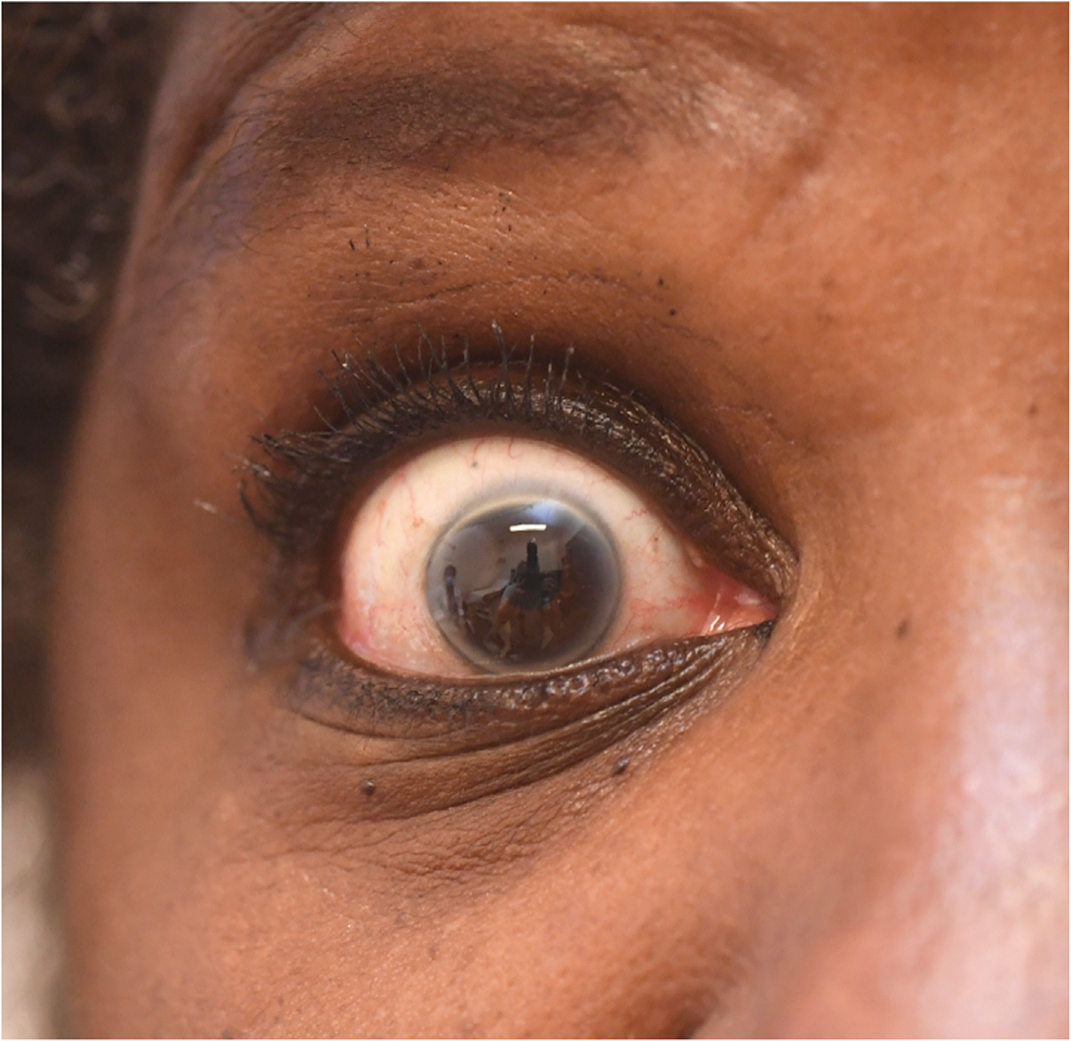
Bilateral Corneal Arcus Arcus is caused by lipid deposits in the peripheral cornea. In individuals age <45 years, it is specific for familial hypercholesterolemia, whereas it is common and nonspecific in the elderly.

**Figure 3:**
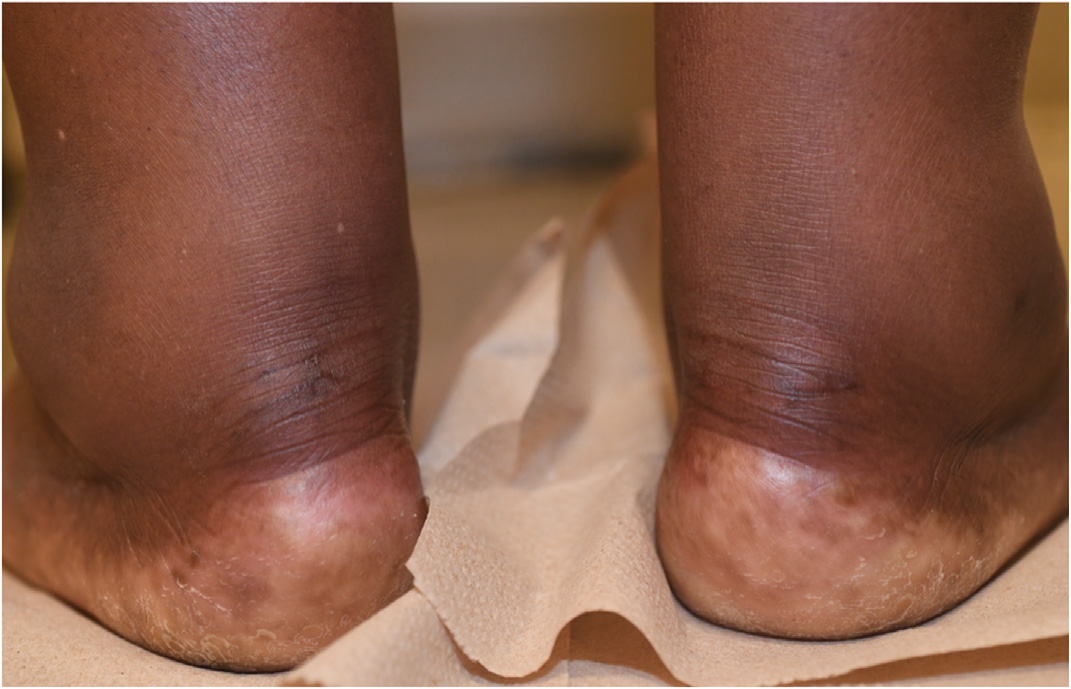
Achilles Tendon Xanthoma Xanthomas consist of lipid deposits in the dermis, subcutaneous tissue, and tendons, and are pathognomonic for familial hypercholesterolemia.

Her most recent physical examination at age 67 years was significant for arcus (Figure 2), and xanthomas over both Achilles’ tendons (Figure 3), patellar tendons (Supplemental Figure 1), and interdigitally (Supplemental Figure 2).

## Past Medical History

Accompanying multivessel coronary artery disease, angina, and peripartum cardiomyopathy, as noted in the previous text, her medical history includes former smoking, carotid artery stenosis, ophthalmic stenosis, right renal stenosis, atrial fibrillation, Graves’ disease, essential hypertension, prediabetes, Achilles tendinitis, impaired ambulatory status, fistula-related chronic regional pain syndrome, multiple sclerosis, and anemia.

## Differential Diagnosis

The differential diagnosis for severe hypercholesterolemia with total cholesterol >1,000 mg/dL is quite narrow. Conditions associated with secondary hypercholesterolemia, such as hypothyroidism, do not generally cause such severe hypercholesterolemia. Her lipid levels together with examination findings of early-onset arcus and xanthomata, along with premature coronary artery disease and family history, are most compatible with a HoFH diagnosis. Rarely, lipoprotein X because of biliary obstruction can present with total cholesterol levels >1,000 mg/dL. However, no biliary obstruction was observed in this case.

## Investigations

Genetic testing included sequencing and deletion duplication analysis of *LDLR, APOB,* proprotein convertase subtilisin-kexin type 9 (*PCSK9*)*,* and *LDRLRAP1*. Testing found 2 variants in the *LDLR* gene: c.1846-1G>A and c.907C>T.

## Management

**Figure 4:**
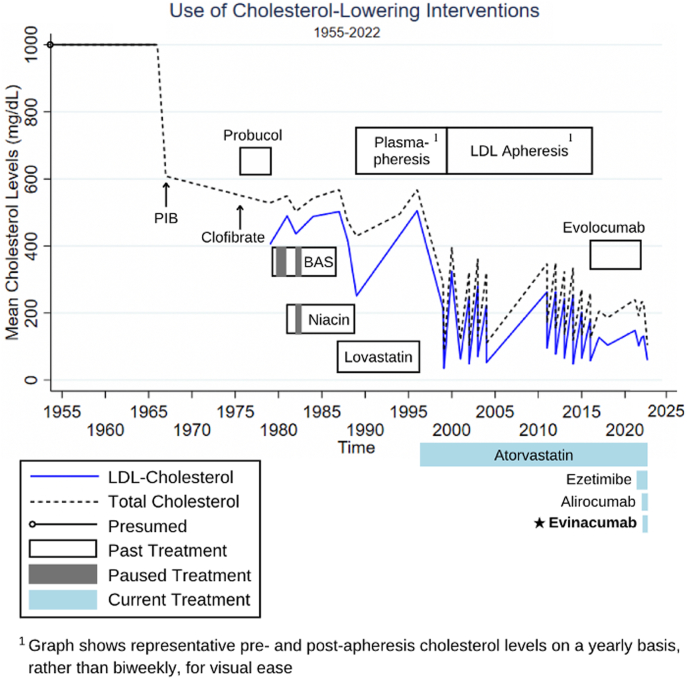
Cholesterol Levels and Treatments, 1955 to 2022 Standard lipid-lowering treatments were insufficient; the most recent addition of evinacumab helped achieve low-density lipoprotein (LDL) cholesterol <70 mg/dL. BAS = bile acid sequestrant; PIB = partial ileal bypass.

**Figure 5:**
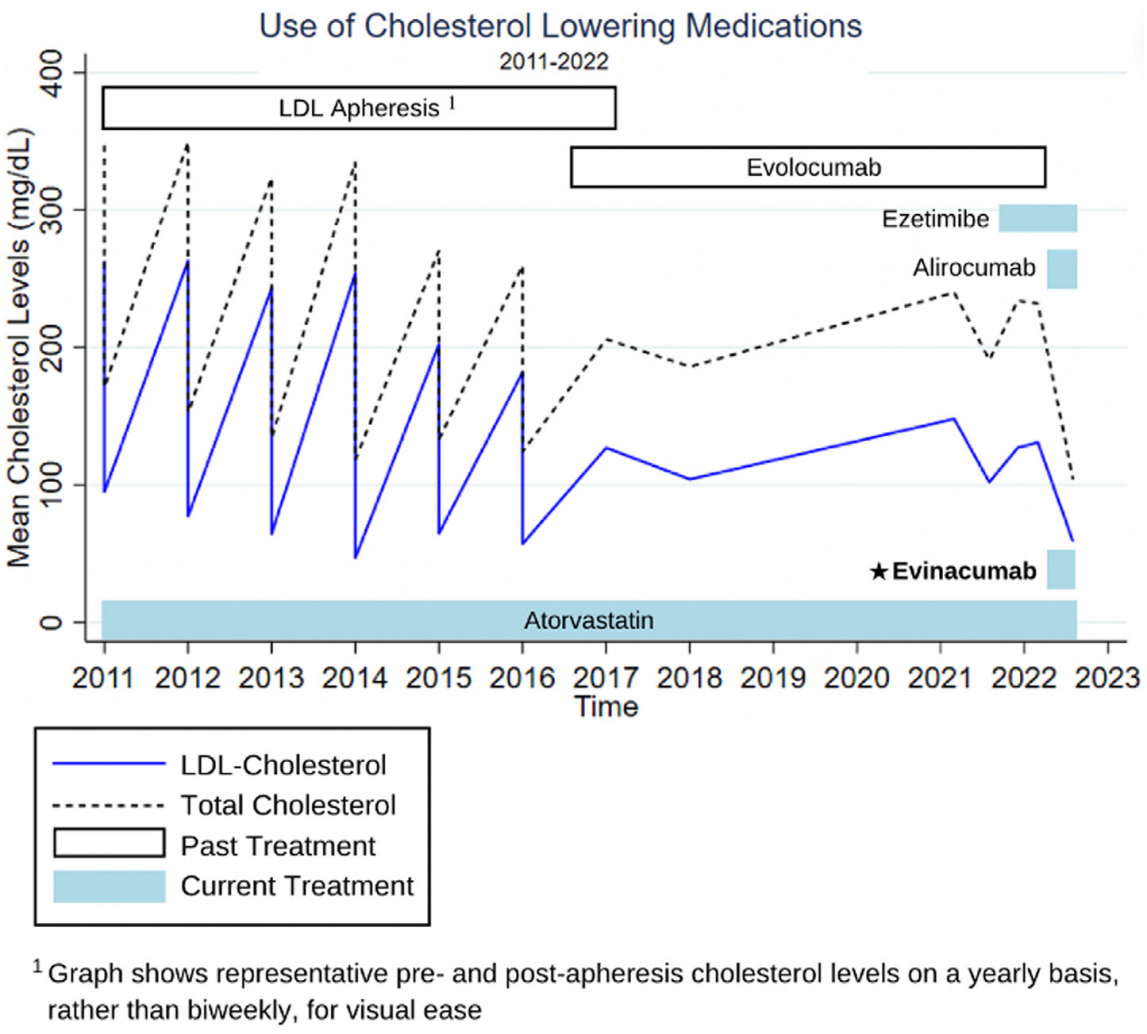
Cholesterol Levels and Treatments, 2011 to 2022 An expanded view of recent low-density lipoprotein (LDL) cholesterol reductions.

In addition to being postileal bypass and on a low-fat diet, the patient was continued on atorvastatin 80 mg and ezetimibe 10 mg daily. Alirocumab 150 mg every 14 days was substituted for evolocumab (based on a change in insurance preference), and evinacumab 15 mg/kg of body weight monthly was added at age 67 years. Before treatment, her LDL-C was 131 mg/dL; afterward, it was 59 mg/dL. Her follow-up over 67 years demonstrates a mean 85% LDL-C reduction from combination therapy (Figures 4 and 5).

## Discussion

HoFH prevalence is estimated to be 1 in 170,000 to 300,000.^1^ Clinical guidelines for diagnosis from the American Heart Association include LDL-C ≥400 mg/dL and 1 or both parents with clinically diagnosed familial hypercholesterolemia, or LDL-C >560 or >400 mg/dL with aortic valve disease or xanthomata before age 20 years.^2^ Genetic testing can also aid in diagnosis. Left untreated, HoFH patients typically manifest symptoms of premature atherosclerotic cardiovascular disease (ASCVD) before age 20 years and experience fatal myocardial infarctions or strokes in their 30s.

HoFH is refractory to standard lipid-lowering agents, including statins, because of limited residual low-density lipoprotein receptor activity. In our patient’s case, despite dietary modification, ileal bypass, and various medications, her LDL-C remained ∼400 mg/dL. Plasmapheresis or more refined techniques of lipoprotein apheresis lowered LDL-C acutely by ∼80%; however, LDL-C then drifted toward the baseline between treatments, leading to a lower time-averaged response.

Surgical approaches (ie, PIB, liver transplantation) are less commonly used because of complications and adverse effects. Our patient’s PIB at age 12 years resulted in ∼50% total cholesterol reduction. It likely explains her overall positive clinical course and later-onset coronary symptoms relative to most HoFH patients.

Genetic testing, and PCSK9 and angiopoietin-like 3 inhibitors, have emerged as promising diagnostic tools and therapies, respectively.^3^^,^^4^ HoFH, an autosomal codominant disorder, involves defective LDL receptor structure or function because of loss-of-function variants in *LDLR, APOB,* or *LDLRAP* genes or gain-of-function variants in *PCSK9.* Two pathogenic variants in *LDLR* were identified in our patient.

The most recent addition of evinacumab to statin, ezetimibe, and PCSK9 inhibitor therapy provided superior LDL-C–lowering efficacy. Alirocumab and evolocumab are monoclonal antibodies that bind and inhibit circulating PCSK9; evinacumab is a monoclonal antibody against angiopoietin-like 3. Evinacumab has a unique mechanism of action that is independent of the LDL receptor; it reduces LDL-C levels through up-regulation of lipoprotein lipase and endothelial lipase.^5^^,^^6^ Limitations include its cost and intravenous route of administration, potentially leading to access challenges. The goal of therapy is to achieve LDL-C levels <100 mg/dL in adults without ASCVD, <70 mg/dL in ASCVD patients, and <55 mg/dL in very-high-risk ASCVD patients.^7^

## Follow-Up

The patient continues to receive evinacumab infusions monthly. Her cholesterol levels have shown continual reductions to LDL-C <70 mg/dL.

## Conclusions

Our patient developed HoFH because of pathogenic variants in the *LDLR* gene. This case highlights tremendous innovation in therapies over the decades. The future of HoFH is bright, but early intervention and aggressive combination therapy are needed to close ongoing treatment gaps. In our patient’s words, “I’ve been going through the history of this disease in one lifetime…it’s been luck meets opportunity meets knowledge.”

## Funding Support and Author Disclosures

Dr Martin has received research support from the American Heart Association (20SFRN35380046, 20SFRN35490003, 878924, 882415), PCORI (ME-2019C1-15328), National Institutes of Health (R01AG071032, P01 HL108800), the David and June Trone Family Foundation, Pollin Digital Health Innovation Fund, the PJ Schafer Cardiovascular Research Fund, Sandra and Larry Small, CASCADE FH, Apple, Google, and Amgen outside of this work; has received consulting fees from Amgen, AstraZeneca, Dalcor, Esperion, iHealth, Kaneka, Novartis, Novo Nordisk, Sanofi, and 89bio outside of this work; and is a coinventor of the Martin/Hopkins method of low-density lipoprotein cholesterol calculation, for which the patent application has been abandoned by Johns Hopkins university to enable use without intellectual property restrictions. All other authors have reported that they have no relationships relevant to the contents of this paper to disclose.
