# Supplementary material for: Homozygous Familial Hypercholesterolemia: Luck Meets Opportunity Meets Knowledge
Source: JACC Case Rep. 2022 Nov 8;4(23):101666. doi: 10.1016/j.jaccas.2022.10.005 (PMC9730161; doi:10.1016/j.jaccas.2022.10.005)
Supplement: Supplemental Figure 1–2 [file mmc1.docx]

**Supplemental Appendix**


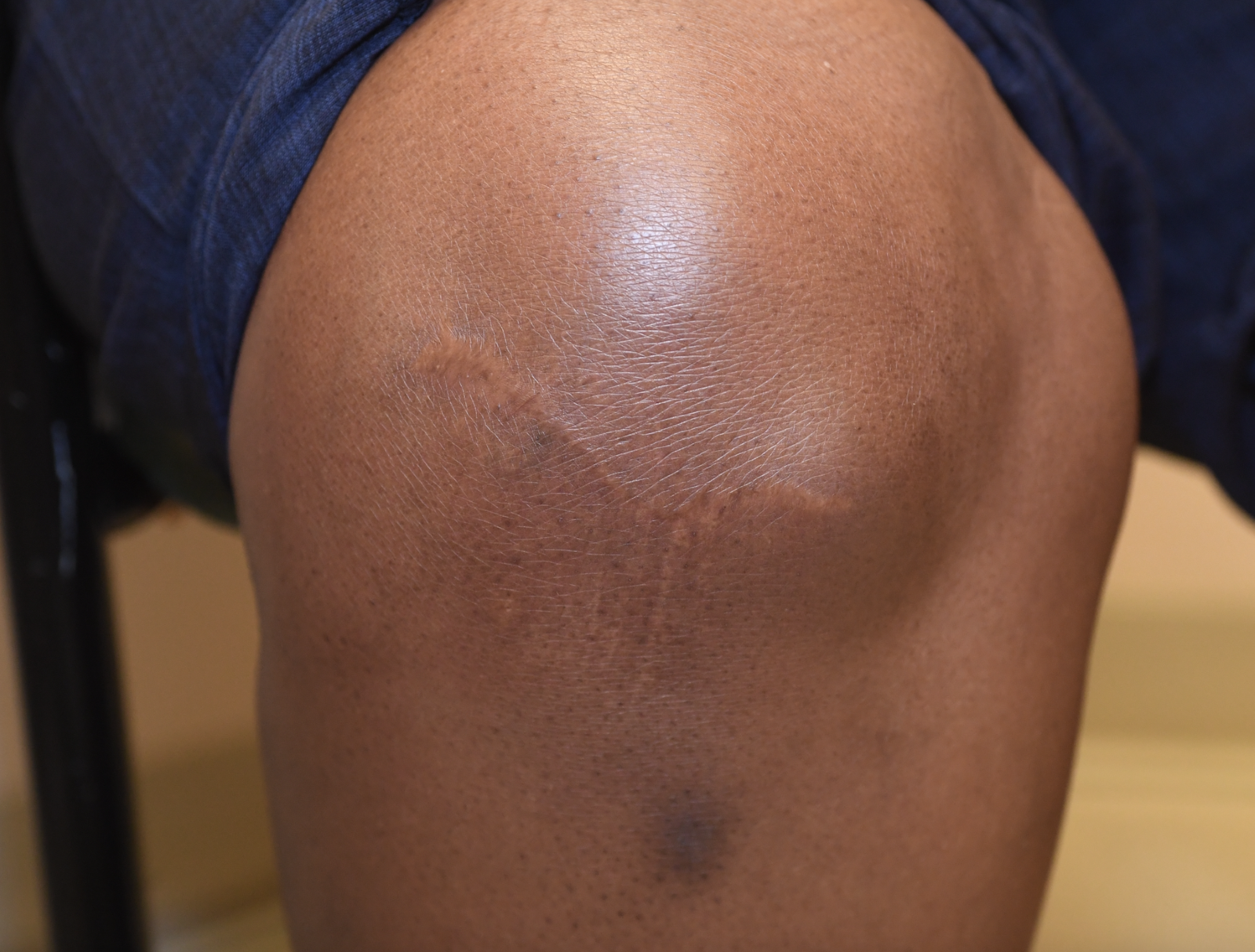


**Supplemental Figure 1. Patellar Tendon Xanthoma.** This xanthoma was surgically resected at the age of 12 years.


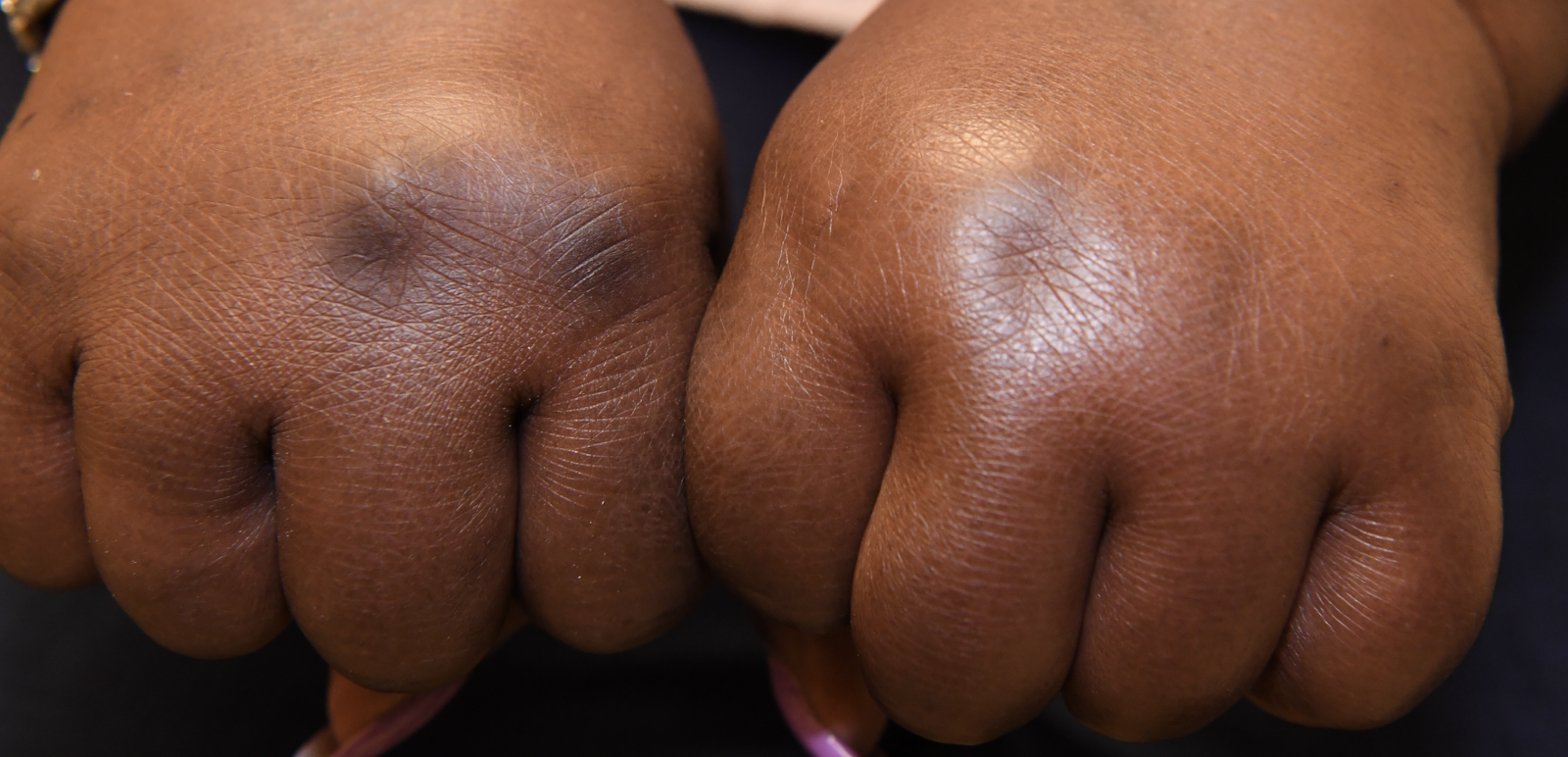


**Supplemental Figure 2. Interdigital Xanthomas.** Xanthomas located between the digits are pathognomonic for familial hypercholesterolemia.
